# Methylglyoxal and Glyoxal as Potential Peripheral Markers for MCI Diagnosis and Their Effects on the Expression of Neurotrophic, Inflammatory and Neurodegenerative Factors in Neurons and in Neuronal Derived-Extracellular Vesicles

**DOI:** 10.3390/ijms20194906

**Published:** 2019-10-03

**Authors:** Mohamed Haddad, Morgane Perrotte, Mohamed Raâfet Ben Khedher, Clément Demongin, Aurélie Lepage, Tamás Fülöp, Charles Ramassamy

**Affiliations:** 1 Institut National de Recherche Scientifique – Centre Armand-Frappier Santé Biotechnologie, Laval, QC H7V 1B7, Canada; Mohamed.Haddad@iaf.inrs.ca (M.H.); perrottemorgane@gmail.com (M.P.); Mohamed.BenKheder@iaf.inrs.c (M.R.B.K.); clement.demongin@laposte.net (C.D.); 2 Institute on Nutrition and Functional Foods, Laval University, Quebec City, QC G1V 0A6, Canada; 3 Department of Medicine, Geriatric Division, Research Center on Aging, Sherbrooke University, Sherbrooke, QC J1H 4C4, Canada; Aurelielepage18@gmail.com (A.L.); Tamas.Fulop@USherbrooke.ca (T.F.)

**Keywords:** Alzheimer’s disease, mild cognitive impairment, extracellular vesicles, advanced glycation products, Growth, inflammatory and vascular factors

## Abstract

Methylglyoxal (MG) and glyoxal (GO) are suggested to be associated with the development of neurodegenerative pathologies. However, their peripheral levels in relation to cognitive decline and their effects on key factors in neuronal cells are poorly investigated. The aim of this study was to determine their serum levels in MCI (mild cognitive impairment) and Alzheimer’s disease (AD) patients, to analyze their effects on the neurotrophic and inflammatory factors, on neurodegenerative markers in neuronal cells and in neuronal derived-extracellular vesicles (nEVs). Our results show that MG and GO levels in serum, determined by HPLC, were higher in MCI. ROC (receiver-operating characteristic curves) analysis showed that the levels of MG in serum have higher sensitivity to differentiate MCI from controls but not from AD. Meanwhile, serum GO levels differentiate MCI from control and AD groups. Cells and nEVs levels of BDNF, PRGN, NSE, APP, MMP-9, ANGPTL-4, LCN2, PTX2, S100B, RAGE, Aβ peptide, pTau T181 and alpha-synuclein were quantified by luminex assay. Treatment of neuronal cells with MG or GO reduced the cellular levels of NSE, PRGN, APP, MMP-9 and ANGPTL-4 and the nEVs levels of BDNF, PRGN and LCN2. Our findings suggest that targeting MG and GO may be a promising therapeutic strategy to prevent or delay the progression of AD.

## 1. Introduction 

Dementia affects more than 50 million people and this number will triple in 30 years in the absence of preventive strategy and new effective treatments [1]. Alzheimer’s disease (AD), the most common form of dementia (60–70% of all cases), is clinically characterized by cognitive decline and behavioral disturbances with a complex and heterogeneous pathophysiology [2]. In addition to both neuropathological hallmarks of AD, the accumulation of the amyloid beta peptide (Aβ) in senile plaques and the neurofibrillary tangles (NFTs), there are also imbalances in many cerebral proteins such as neurotransmitters, neuroprotective, neurotrophic and inflammatory factors [3,4,5,6,7]. To date, the diagnosis of AD represents a late stage of the disease and is associated with irreversible brain damage [8]. Therefore, the identification of early AD biomarkers is crucial to prevent or delay the onset of the disease and to slow the progression of this brain disorder. It has been shown that the diagnosis of AD at the earlier stage may prolong survival [9]. 

Mild cognitive impairment (MCI) is considered as a transitional state between normal aging and dementia associated with memory and cognitive impairment (greater of what is observed for aged individuals) that does not interfere notably with activities of daily life [10]. The prevalence of MCI is estimated to be between 15% and 20% beyond 60 years [11,12,13]. MCI was associated with an increased risk of progression to dementia and particularly AD [14]. The physiopathology of MCI remains unclear, but it is suggested that metabolic, neurologic and psychiatric disorders largely contribute to this early cognitive impairment [15]. Currently, the diagnosis of MCI is based on cognitive tests, the determination of the ratio p-tau/Aβ_42_ in cerebrospinal fluid (CSF) and neuroimaging analysis [16]. However, the routine application of these criteria is relatively limited and difficult because of the high costs of imaging, the invasive nature of CSF collection and the clinical heterogeneity of the MCI patients. Therefore, there is a need to identify blood-based biomarkers related to neurodegenerative process and associated with MCI stage that enable an early diagnosis of the disease with reduced costs. 

Advanced glycation end products (AGEs) are heterogeneous class of molecules termed by the interaction of reducing sugars or dicarbonyl compounds such as methylglyoxal (MG) or glyoxal (GO) with amino groups in proteins through a series of Maillard reactions [17]. Interestingly, increased levels of AGEs were reported in brains of AD patients and were also found to be associated with the amyloid plaques and NFTs [18,19,20,21]. MG and GO are the precursors of many AGEs such as *N*-(1-carboxymethyl)-*L*-lysine (CML), glyoxal-lysine dimer (GOLD), *N*-(carboxyethyl)-*L*-lysine (CEL) and methylglyoxal-lysine dimer (MOLD). MG and GO are two reactive intermediates of cellular metabolism produced by several different pathways [22,23]. Many studies have reported the capacity of these reactive intermediates to induce cellular damage and contribute to the pathogenesis of many neurodegenerative diseases. For instance, increased intracellular reactive oxygen species production, tau hyperphosphorylation, mitochondrial dysfunction [24,25,26], expression of cleaved caspase-3, apoptosis and decreased Bcl-2/Bax ratio were observed in neuronal cells following MG treatment [27]. The intracerebroventricular (ICV) administration of MG induced tau hyperphosphorylation and caused hippocampal damage and memory impairment in mice [28]. Elevation of the levels of MG in streptozotocin-induced diabetic rats was associated with cognitive impairments in memory and learning [29]. ICV administration of MG induces cognitive deficit and neurochemical alterations in the hippocampus of rat brain [30]. Recently, we demonstrated that the serum levels of CML, a by-product of MG and GO, increased early in AD and correlated with the cognitive scores of Mini Mental State Examination (MMSE) and Montreal Cognitive Assessment (MoCA) tests [31]. 

Therefore, AGEs precursors (MG and GO) could be considered as strong candidates for MCI diagnosis. However, neurotoxic effects of these metabolites especially on the neuroprotective, neurotrophic factors, inflammatory and neurodegenerative markers remain to be determined. 

Extracellular vesicles (EVs) are whole membrane-bound vesicles such as exosomes and microvesicles. EVs play a fundamental role in intercellular communication by transferring heterogeneous biological materials including signaling factors, misfolding proteins, nucleic acid, and regulatory molecules [32]. Many cells in the central nervous system such as neurons, astrocytes and microglia release EVs [33,34,35]. Several studies have suggested the implication of EVs in AD and showed that these vesicles contain a variety of implicated proteins in the disease [36,37,38]. However, the effects of MG and GO on the levels of neuroprotective, neurotrophic factors, inflammatory and neurodegenerative markers in neuronal derived-EVs (nEVs) are still unknown. 

The first aim of the present work was to measure the serum levels of MG and GO in controls, MCI and AD patients. The second aim was to study the effects of these AGEs precursors on the neuronal expression of neuroprotectors and neutrophic factors such as BDNF (Brain-Derived Neurotrophic Factor), progranulin (PRGN), NSE (neuron specific enolase), APP (amyloid precursor protein) and MMP-9 (matrix metallopeptidase 9), proteins implicated in the inflammatory process (ANGPTL-4 (Angiopoietin Like 4), lipocalin-2 (LCN2), pentraxin-2 (PTX2), S100B (calcium binding protein B), AGEs (CML), and RAGEs (receptor for advanced glycation end products)), and neurodegenerative markers (Aβ peptide, pTau T181, and alpha synuclein). Finally, the neuronal levels of these proteins were compared to their nEVS levels. This study addressed a complete panel of the effects of MG and GO on neuronal cells. 

## 2. Results

### 2.1. Main Characteristics of Study Population

The clinical characteristics and glucose levels of controls, MCI and AD groups are shown in Table 1. The mean age, and the clinical scores of MMSE and MoCA were significantly different among controls, MCI and AD patients. MMSE and MoCA scores were not available for LS patients, probably because they were unable to answer or complete the questionnaire due to their severe cognitive impairment. Although blood glucose levels in moderate AD patients were higher compared to controls, the values remain in the physiological range of circulating levels. 

### 2.2. MG and GO Serum Levels in Control Subjects, in MCI and AD Patients 

**Figure 1:**
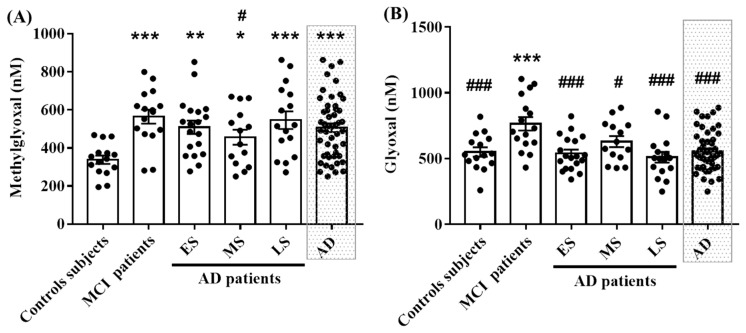
Levels of MG and GO in serum from control, MCI and different AD groups. MG (**A**) and GO (**B**) serum levels are expressed in nM. Each point represents the value obtained from one patient or control subject. The difference between groups was analyzed with one-way ANOVA followed by the LSD post hoc test. Values are mean ± S.E.M with * *p* < 0.05, ** *p* < 0.01, *** *p* < 0.001 versus control subjects. ^#^ *p* < 0.05, ^###^ *p* < 0.001 versus MCI patients.

The results show that MG levels were significantly higher in MCI and AD patients compared to the control subjects. Moreover, MG levels in MS group of AD patients were lower than in the MCI group (Figure 1A). The GO levels were significantly increased only in MCI patients compared to the control and AD’s groups (Figure 1B). 

**Figure 2:**
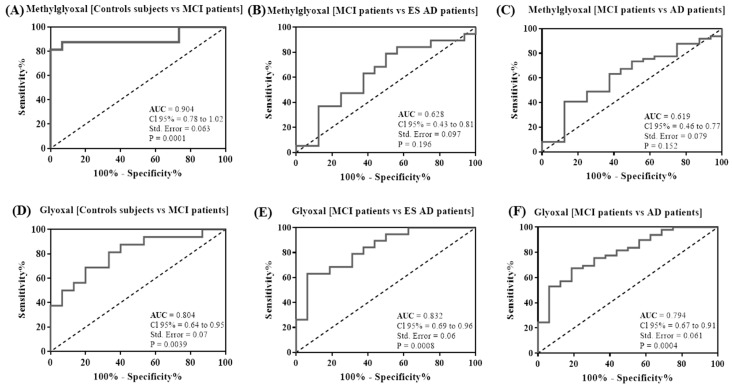
Receiver operating characteristic (ROC) curve analysis. The plots represent the performance of MG and GO serum levels to differentiate MCI patients to control subjects (**A**,**D**) and to early AD patients (**B**,**E**) and all AD patients (**C**,**F)**. Area under the curve (AUC) values, 95% confidence intervals (CI 95%), standard error (Std. Error) and *p* values are indicated on the curve.

The ability of the MG and GO serum levels to distinguish control subjects from MCI and AD groups was assessed using the ROC analysis. The levels of MG and GO provide a fair classification of the control group and MCI patients with an area under the curve (AUC) of 0.904 (95% CI: 0.78–1.02, *p* = 0.0001) and 0.804 (95% CI: 0.64–0.095, *p* = 0.0039), respectively (Figure 2A,D). The optimal cut-off value of MG and GO levels to differentiate MCI patients from control subjects was 463.2 nM, with 87.5% sensitivity and 93.33% specificity, for MG and 652.2 nM, with 68.75% sensitivity and 80% specificity, for GO (Table 2). To distinguish MCI from ES or all AD patients, ROC curves for MG levels had an AUC of 0.628 (95% CI: 0.43–0.81, *p* = 0.196) and 0.619 (95% CI: 0.46–0.77, *p* = 0.152), respectively, indicating that MG levels have low classification accuracy (Figure 2B,C). Interestingly, when ROC curves were applied for GO levels for these same groups, we obtained an AUC of 0.832 (95% CI: 0.69–0.96, *p* = 0.0008) and 0.794 (95% CI: 0.67–0.91, *p* = 0.0004), respectively, indicating that GO levels have high classification accuracy (Figure 2E,F). The optimal cut-off value of GO levels to predict MCI patients from ES or all AD patients was <588.6 nM, with 68.42% sensitivity and 81.25% specificity, and <605 nM, with 67.35% sensitivity and 81.25% specificity, respectively (Table 2).

### 2.3. Effects of MG and GO on SK-N-SH cells Viability

**Figure 3:**
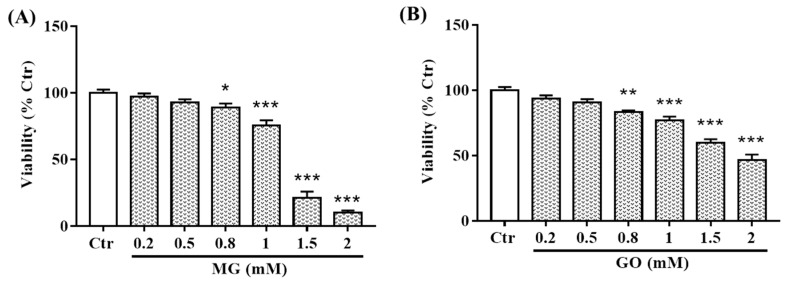
Effects of MG and GO on the survival of SK-N-SH neuronal cells. Cells were treated for 24 h with different concentrations (0.2–2 mM) of MG (**A**) and GO (**B**). Cell survival was analyzed by Resazurin assay. The results are expressed as a percentage of control, non-treated cells (considered as 100%). Values are mean ± S.E.M from at least three separate experiments performed in sextuplicate in each group with * *p* < 0.05, ** *p* < 0.01, *** *p* < 0.001 versus control cells. Data groups were compared with one-way ANOVA followed by the Dunnett’s post hoc test.

The neurotoxicity of MG and GO was performed on SK-N-SH cells. Our results show that, after 24 h of treatment, MG and GO were not toxic until 0.8 mM (Figure 3). The concentration of 0.5 mM was thus selected for further experiments for both MG and GO. 

### 2.4. Effects of MG and GO on the Size and Density of Extracellular Vesicles Released by the SK-N-SH Neuronal Cells 

**Figure 4:**
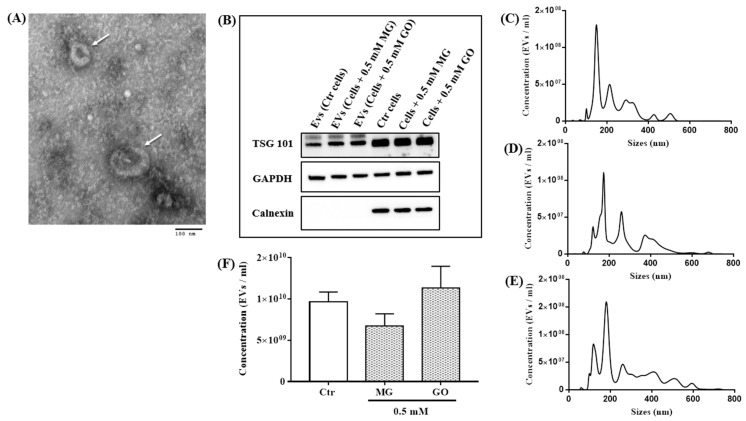
Characterization of nEVs isolated from controls and treated cells. The TEM image revealed the spherical morphology of the isolated particles (White arrow), bar = 100 nm (**A**). Expressions of EVs marker (TSG101), the presence of GAPDH and the negative control (Calnexin) analyzed by Western blot on protein lysates from EVs and SK-N-SH cells (**B**). Concentrations and size of isolated EVs from control cells (**C**), MG-treated cells (**D**) and GO-treated cells (**E**) were analyzed by NTA using Nanosight NS300 system. The concentrations of isolated EVs from control and treated cells with (MG and GO) were not different (**F**). Values are mean ± S.E.M (from three separate experiments) and data groups were compared with one-way ANOVA followed by the Dunnett’s post hoc test.

Neuronal SK-N-SH cells derived-EVs (nEVs) were isolated as previously described [31]. Different approaches were used to characterize EVs. TEM images revealed that the isolated EVs were surrounded with a lipid layer producing a cup-shaped morphology (Figure 4A). In addition, Western blot analysis revealed the presence of EVs marker (TSG101), the presence of GAPDH and the absence of calnexin (a negative EVs marker) (Figure 4B). Size distribution and concentration of nEVs were measured by Nanosight NS300. Our results show that the size distribution of nEVs was not different in control and treated cells with the majority of isolated nEVs being between 50 and 400 nm (Figure 4C–E). Finally, the concentrations of total isolated nEVs were also similar in controls (9.6 × 10^9^ ± 1.18 × 10^9^) and in cells treated with MG (6.7 × 10^9^ ± 1.4 × 10^9^) and GO (1.13 × 10^10^ ± 2.6 × 10^9^) (Figure 4F). 

### 2.5. Effects of MG and GO Treatments on the Levels of BDNF, PRGN, NSE, APP and MMP-9 in Cells and in nEVs 

The effects of MG and GO on the levels of BDNF, PRGN, NSE, APP and MMP-9 in SK-N-SH cells and in nEVs were analyzed following 24 h of treatment. 

**Figure 5:**
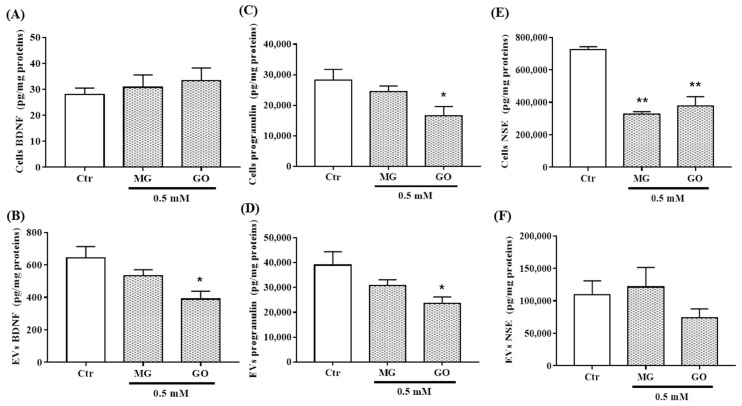
BDNF, PRGN and NSE levels in nEVs and in SK-N-SH cells exposed or not to MG and GO. The level of each marker was normalized using total protein concentration. The bar graphs showBDNF, PRGN and NSE levels in SK-N-SH cells (**A**,**C**,**E**) and in/on EVs (**B**,**D**,**F**). Values are mean ± S.E.M (from three separate experiments) and data groups were compared with one-way ANOVA followed by the Dunnett’s post hoc test. * *p* < 0.05, ** *p* < 0.01 versus control cells.

**Figure 6:**
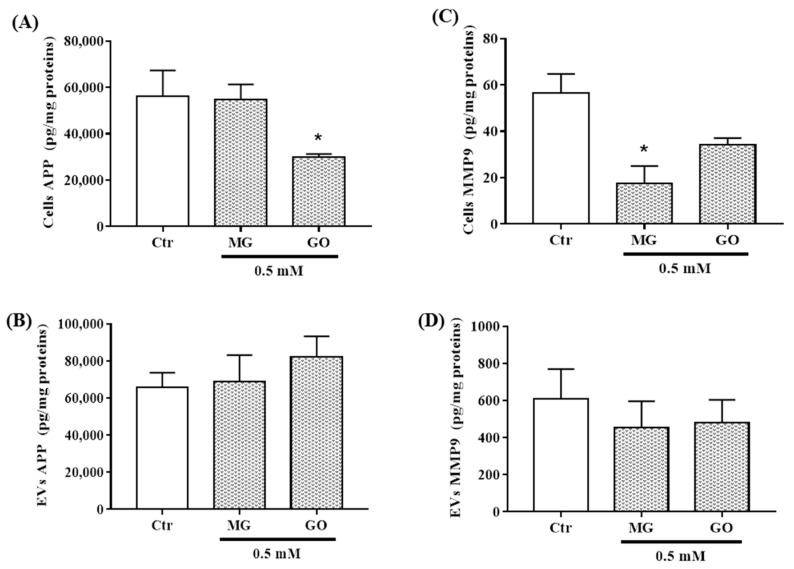
APP and MMP9 levels in nEVs and in SK-N-SH exposed or not to MG and GO. The level of each marker was normalized using total protein concentration. The bar graphs show APP and MMP9 levels in SK-N-SH cells (**A**,**C**) and in nEVs (**B**,**D**). Values are mean ± S.E.M (from three separate experiments) and data groups were compared with one-way ANOVA followed by the Dunnett’s post hoc test. * *p* < 0.05 versus control cells.

BDNF was not affected by MG and GO while PRGN, NSE, APP and MMP-9 levels were differently reduced by MG and/or GO (Figure 5A,C,E and Figure 6A,C). 

Interestingly, neuronal cells can release and transfer these proteins via nEVs. We found that MG or GO treatment reduced the levels of BDNF and PRGN in nEVs without affecting NSE, APP and MMP-9 (Figure 5B,D,E and Figure 6B,D). 

### 2.6. Effects of MG and GO Treatments on the Levels of ANGPTL-4, LCN2, PTX2, S100B and RAGE in Cells and nEVs

**Figure 7:**
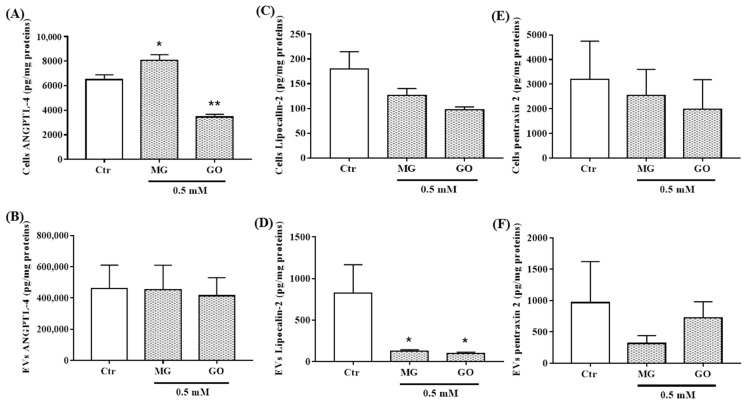
ANGPTL-4, LCN2 and PTX2 levels in nEVs and in SK-N-SH cells exposed or not to MG and GO. The level of each marker was normalized using total protein concentration. The bar graphs show ANGPTL-4**,** LCN2 and PTX2 levels in SK-N-SH cells (**A**,**C**,**E**) and in nEVs (**B**,**D**,**F**). Values are mean ± S.E.M (from three separate experiments) and data groups were compared with one-way ANOVA followed by the Dunnett’s post hoc test. * *p* < 0.05, ** *p* < 0.01 versus controls cells.

AGEs are known to induce neuroinflammation. The effects of MG and GO on the expression and secretion of inflammatory proteins remain to be studied. We analyzed their effects on the neuronal level of proteins involved in inflammation such as ANGPTL-4, LCN2, PTX2, S100B and RAGE. We found that only the levels of ANGPTL-4 were affected by MG and GO with a differential effect between MG and GO (Figure 7A,C,E and Figure S1A,C). We also showed that SK-N-SH neuronal cells can propagate these proteins through nEVs. MG and GO greatly decreased the release of LCN2 in nEVs but did not modify the nEVs content of other proteins (Figure 7B,D,F and Figure S1B,D). 

### 2.7. Effects of MG and GO Treatments on the Levels of Aβ_1-40_, Aβ_1-42_, pTau T181 and α-Synuclein in Cells and nEVs 

To determine whether MG and GO treatments affect the expression of neurodegenerative markers, levels of Aβ peptide, pTau T181 and alpha synuclein were determined in cells and in nEVs. We showed that cells treated with nontoxic dose of MG and GO did not affect the neuronal levels of Aβ_1-40_ (Figure S2A) as well as neuronal and nEVs levels of Aβ_1-42_ (Figure S2C,D), alpha-synuclein (Figure S2E,F) and pTau (Figure S3). Aβ_1-40_ was not detected in nEVs, suggesting that Aβ_1-40_ is secreted at very low level in nEVs (Figure S2B). 

### 2.8. Effects of MG and GO on the Levels of the AGE Product CML and on the Deglycase Enzyme DJ-1

**Figure 8:**
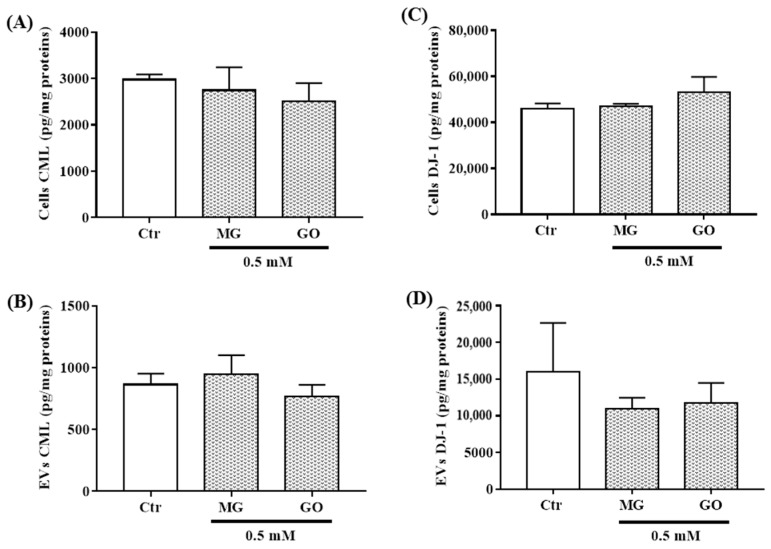
CML and DJ-1 levels in nEVs and in SK-N-SH cells exposed or not to MG and GO. The level of each marker is normalized using total protein concentration. The bar graphs show CML (**A**,**B**) and DJ-1 levels (**C**,**D**) in SK-N-SH cells (**A**,**C**) and nEVs (**B**,**D**). Values are mean ± S.E.M (from three separate experiments) and data groups were compared with one-way ANOVA followed by the Dunnett’s post hoc test.

To investigate whether the effects observed following the MG and GO treatments were mediated through the AGEs formation, we evaluated the levels of CML, a by-product of MG and GO, and DJ-1, a deglycation protein in neuronal cells and in nEVs. Our results show that neither MG nor GO modified the expressions of CML and DJ-1 (Figure 8). These results suggest that the effects observed following the treatments of MG and GO are not related to AGEs formation. 

## 3. Discussion 

Glycation of proteins is involved in the pathophysiology of AD through both extensive protein cross-linking and oxidative stress [39,40,41,42]. In the present study, we analyzed the variations of peripheral levels of two reactive precursors of AGEs with respect to cognitive decline, in MCI and in AD from the early to the late stage of AD. We showed that the levels of MG in serum were higher in MCI patients than in control subjects. ROC analysis demonstrated that its serum levels have a higher probability to differentiate MCI patients from controls but not from AD patients. Interestingly, the serum levels of GO can differentiate controls from MCI and AD patients. MG and GO are known to be present in the brain but their effects on some key target proteins involved in the pathophysiology of various neurodegenerative diseases such as AD remain to be studied. We found that both AGEs precursors, at nontoxic concentration, reduced the neuronal level of NSE with no effect on BDNF, PTRX-2, LCN-2, DJ-1, neurodegenerative markers and CML. GO decreased the levels of PRGN, APP, and ANGPL-4, while MG reduced and induced the expressions of MMP-9 and ANGPL-4, respectively. 

nEVs are known to contribute to the progression of AD [43,44]. We found that MG and GO differently affect the content of some protein cargoes in/on nEVs. Our data show that MG and GO greatly reduced the release of LCN-2 by neuronal cells in/on nEVs. BDNF and PRGN in/on nEVs were reduced in the presence of GO. Neither MG nor GO modified the release of NSE, APP, MMP9, AGNTL-4, PTX-2, DJ-1, Aβ, pTau and CML in nEVs. 

MG is known to be one of the most potent precursors of AGEs and its presence is correlated with increase of oxidative stress in AD. Many studies strongly suggest that MG and MG-derived AGEs may play a key role in the etiopathogenesis of AD. Indeed, MG induced hyperphosphorylation of tau and post-translational modification of neurofilanents and can also decrease PP2 [45,46]. However, the levels of MG in biofluids are poorly investigated. In CSF, the levels of MG were found to be higher in AD and were inversely associated to the MMSE scores [47]. In serum, Beeri et al. previously demonstrated that higher levels of MG were associated with a faster rate of cognitive decline [48], with lower memory and reduced grey matter volume in elderly people [49]. In line with these observations, our data show higher MG and GO serum levels in MCI patients. These clinical studies suggest that MG and GO could easily be used in medical analysis routinely. In serum, the elevation of MG and GO levels could be from different sources. In plasma, the conversion of aminoacetone to MG and H_2_O_2_ is insured by the semicarbazidesensitive amine oxidase (SSAO), an enzyme that can be detected in cell membranes and plasma [50]. Accordingly, SSAO activity was shown to be elevated in plasma from AD patients [51,52]. Recently, the circulating SSAO activity was shown to be associated to the alteration of the blood brain barrier observed during the progression of AD [53]. 

Glycation of circulating proteins and the formation of AGEs by non-enzymatic reaction may also contribute to MG and GO production [54]. Moreover, MG and GO could be released from healthy or injured cells, or supplied exogenously by food intake [55]. On the other hand, the detoxification of MG and GO is dependent on the glyoxalase system, which is composed of two enzymes, glyoxalase-1 and -2 (GLO-1 and GLO-2). This detoxification system requires the antioxidant glutathione (GSH) to form hemithioacetal then S-lactoylglutathione. GLO-2 catalyzes the transformation of *S*-lactoylglutathione into D-lactate and recycling GSH [56]. However, the decrease of circulating GSH in AD [57,58] could limit the glyoxalase activity.

In the human brain, the protein and RNA expressions as well as the activity of GLO-1 in astroglia cells and neurons were found to be elevated before 55 years of age and then gradually decreased. This increase may represent a compensatory mechanism against high dicarbonyl levels and the accumulation of AGEs with aging [59]. In the AD brain, GLO-1 RNA levels and enzymatic activity were upregulated in the early stages of the disease but gradually downregulated in the middle and late stages of the disease [60]. Therefore, the number of AGE-positive neurons increases with the progression of the disease. MG and GO could thus accumulate in neurons with the progression of the disease and particularly in endosomal and lysosomal vesicles of pyramidal neurons in the hippocampus, the dentate gyrus, and in different cortical and entorhinal layers [61]. 

The accumulation of MG and GO is associated with free radicals production, the increase of the activity of prooxidant enzymes, the decrease of antioxidant activities, and mitochondrial dysfunction, leading to cell death [62,63]. These dicarbonyls are able to increase the production of Aβ, oligomers and protofibrils as well as the sizes of aggregates [64,65] and modulate several signaling pathways that play a causative role in AD development [66]. Neurons which show diffuse cytosolic AGEs immunoreactivity also contain hyperphosphorylated tau [18]. 

We studied the effects of MG and GO on the expression of some proteins involved in growth factors, inflammation and on the repair of glycated proteins in neuronal cells. These proteins also play a prominent role in modulating cognition and memory by promoting neuronal survival and development and by regulating synaptic plasticity [3,4,67,68,69,70]. Our data demonstrate that MG or GO, by decreasing the expression of NSE, PRGN, MMP-9 and APP, can reduce the neurotrophic properties, neuritic growth, synaptogenesis, anti-inflammatory activity, and ability of neurons to degrade Aβ, as well as modify the lysosome regulatory properties of neuronal cells [71,72,73,74]. In the brain, ANGPTL-4 is mainly expressed by reactive astrocytes but its regulation in neuronal cells is poorly described. We found that MG and GO differently regulate the neuronal expression of ANGPTL-4. Considering that ANGPTL-4 is a secreted protein with important function in the regulation of brain lipid and glucose metabolism, hypoxia-induced factor, inflammatory responses, angiogenesis, vascular permeability and remodeling [75], our results suggest that MG and GO could have an impact on several functions, particularly on vascular properties. 

MG and GO did not have any effect on the levels of other proteins (BDNF, LCN2, and PTX2), also involved in cell survival, inflammatory responses, and insulin sensitivity, cognitive function and oxidative stress [76,77,78,79,80,81]. In contrast to proBDNF, which could be glycated by MG and GO at the free amino groups of Lys, Arg and Cys residues [82], BDNF was not glycated by MG and GO. These results suggest that the targeted proteins are differently vulnerable to glycation induced by MG or GO. Some of them are not modified by nontoxic concentrations of MG or GO. Surprisingly, MG or GO did not modify the level of CML or up regulate the deglycase enzyme DJ-1 [83,84].

Neuronal cells are known to actively secrete nEVs to eliminate or communicate with other cells. EVs contained various protein cargoes that interfere with neuroprotective mechanisms, nerve regeneration, neuronal development or synaptic plasticity [85]. PTRX-2 [86] and DJ-1 [87] were recently shown to be present in nEVs. For the first time, we demonstrated that neuronal cells can release BDNF, NSE, LCN-2 and MMP-9 through nEVs. 

Thus, another aim of our study was to decipher the role of MG and GO on the release and transfer of these proteins by neuronal cells. We showed that MG and GO did not modify the release of PTRX-2, APP, MMP-9, ANGTL-4, DJ-1, Aβ, pTau and CML in nEVs but reduce the secretion of BDNF, PRGN, and LCN-2 in nEVs. These protein cargoes could be inside or associated to the surface of nEVs. These reductions were not the consequences of the abnormal formation and/or release of nEVs as the quantity and the size distribution of EVs were similar following different treatments. These results suggest that neuronal cells reduce the transfer of some proteins related to neurotrophic, neurogenesis or inflammation (BDNF, PRGN, and LCN-2) to other cells to counteract the effects of MG or GO. It is interesting to note that concentrations of BDNF, PRGN, MMP-9, ANGTL-4, LCN-2, and S100B were much higher in nEVs than their cellular levels, while the concentrations NSE, CML and DJ-1 were lower in nEVs.

MG and GO, at nontoxic concentrations, did not alter cells or nEVs levels of some neurodegenerative markers (Aβ peptide, pTau, and alpha synuclein) and some proinflammatory markers (S100B and RAGE) (Figures S1–S3). These observations could be explained by the fact that the concentration of dicarbonyls compounds used in this study did not increase the formation of AGEs such as CML which are implicated in the modulation of neurodegenerative and proinflammatory markers. 

MG and GO have the same dicarbonyl structure except the presence of a methyl group on the carbon skeleton of MG. The differential effect between MG and GO may be due to the fact that GO is considered as a poor substrate for the glyoxalase system since it binds to the α-NH_2_ group or to the cysteine residue of GSH [88,89]. Furthermore, GO but not MG can form glycolate which is oxidized to glyoxylate with the release of H_2_O_2_ [90]. GO is also able to react with hydroxyl radical to form a GO radical and this reaction is three times faster than the reaction between MG and hydroxyl radicals [91,92]. The induction of oxidative stress is a possible mechanism that explains the differential effects of MG and GO.

## 4. Materials and Methods

### 4.1. Clinical Study

The present study was performed on serum samples from control subjects, MCI and AD patients recruited from the Memory Clinic of Sherbrooke. The study protocol was approved by the Ethics Committee of the University of Sherbrooke and informed written consent was obtained from all participants and/or their representatives (protocol #2010-21/Fülöp approved 12 Novembre 2010). The AD patients at different stage (early, moderate and last stage) were selected relatively to the criteria of the National Institute of Neurological and Communicative Disorders and Stroke and the Alzheimer’s Disease and Related Disorders Association (NINCDS_ADRDA) and the fourth edition of the Diagnostic and Statistical Manual of Mental Disorders (DSM-IV) published by the American Psychiatric Association in 1984 [93]. Clinical selection of MCI patients was performed depending on cognitive test scores and Pertersen criteria [94]. The healthy control subjects were defined according to the SENIEUR protocol [95]. All selected subjects underwent clinical and neuropsychological evaluation included the MoCA and MMSE [96,97]. Serum samples were obtained from selected subjects after overnight fasting and stored at −80 °C until analysis. 

### 4.2. Serum Methylglyoxal and Glyoxal Measurement by High-Performance Liquid Chromatographic (HPLC)

MG and GO levels were determined from serum by HPLC (Beckman system Gold) with UV-Visible detector according to the authors of [98] with some modifications. One hundred microliters of serum from different groups were incubated with trichloroacetic acid (TCA) (Sigma Aldrich, Foster City, CA, USA) for 10 min. The precipitated protein was removed from the mixture by centrifugation at 12,000 rpm for 15 min at 4 °C. O-Phenylenediamine (O-PD) (10 mM final concentration) (Sigma Aldrich) was added to the supernatant and the mixture was incubated during 24 h at room temperature to derivatize MG and GO. After 10 min of centrifugation at 12,000 rpm, 40 µL of supernatant with known concentration of the internal standard 5-methylquinoxaline (5-MQ) (Sigma Aldrich) were injected into HPLC. The running program was performed with 20% acetonitrile (kept at 100%) for running the samples and 80% methanol used for washing the column and lines with a constant flow rate set at 1 mL/min. The quantification of MG was based on the determination of its derivative compound 2-methylquinoxaline (2-MQ) (Sigma Aldrich) which absorbed at 315 nm. Different concentrations of 2-MQ external standard were used to define the retention time via C18 column (150 mm × 4.6 mm and 5-μm particle size). To determine GO retention time, different concentrations of GO solution (Sigma Aldrich, CA) were incubated with O-PD and injected into the HPLC. 

### 4.3. Neuronal Cells Culture, Treatment and Viability Assay 

SK-N-SH (Human neuroblastoma) cell line from ATCC (Manassas, VA, USA) were cultivated in Eagle’s minimal essential medium (EMEM) supplemented with 10% (*v*/*v*) Fetal Bovine Serum (FBS), 1% sodium pyruvate (1 mM) and antibiotics (100 U/mL penicillin and 100 µg/mL streptomycin) in a humidified incubator at 37 °C with 5% CO_2_ to 80% confluence. SK-N-SH cells were selected in this study because they are brain-derived neuronal cells from human. We previously and repeatedly demonstrated that these cells contain several signaling pathways involved in neuronal functions.

SK-N-SH cells were plated at a density of 2 × 10^4^ cells per well in 96-well plates and incubated for 24 h at 37 °C, and then the media was completely removed and cells were kept in FBS free media. Cells were treated with different concentrations of MG and GO (0.2–2 mM) for 24 h. Cells survival was assessed using the Tox-8 (Resazurin-based) following the manufacturer’s instructions. 

### 4.4. Isolation of Extracellular Vesicles from Culture Media

Neuronal derived-EVs (nEVs) from culture media were isolated as previously described [31]. Briefly, SK-N-SH cells were grown to 80% of confluence, washed with PBS and incubated in FBS free media with MG and GO (0.5 mM) for 24 h. Supernatant were collected from five 125 cm^2^ flasks and centrifuged to remove dead cells and cell debris. The final supernatant was precipitated with the total exosome isolation reagent from (Invitrogen™ by Life Technologies Inc., Carlsbad, CA, USA). The nEVs pellet was resuspended in PBS and stored at −80 °C until analysis. Total proteins from SK-N-SH cells and nEVs were extracted with RIPA buffer (50mM Tris buffer, pH 8, 150 mM sodium chloride, 0.1% sodium dodecyl sulfate, 1% Igepal, 1% sodium deoxycholate, 5 mM EDTA) containing a cocktail of protease and phosphatase inhibitors and were measured using bicinchoninic acid (BCA) assay (Pierce™ BCA Protein Assay Kit, ThermoFisher Scientific, Inc, Waltham, MA, USA). 

### 4.5. Characterization of Extracellular Vesicles

The characterization of nEVs from culture media was based on the analysis of the morphology by transmission electron microscopy (TEM), their sizes distribution by the Nanosight system (NS300), the presence of some EVs markers (TSG101(tumor susceptibility gene 101) and GAPDH (Glyceraldehyde 3-phosphate dehydrogenase)) and the absence of endoplasmic reticulum marker Calnexin by Western blot.

#### 4.5.1. Transmission Electron Microscopy

EVs preparations were mixed with 2% paraformaldehyde (final concentration), and then applied onto Formvar-carbon coated grid. After 5 min, grids were negatively stained with 2% uranyl acetate solution. The excess fluid was removed and grids were observed using HITACHI 7100 TEM at 75 kV and 15,000×-40,000× magnification. 

#### 4.5.2. Nanoparticles Tracking Analysis

The Nanosight NS300 instrument and Nanosight NTA 3.2 Analytical Software (Malvern Instruments Company, Nanosight, and Malvern, United Kingdom) were used for size and concentration analyses of isolated nEVs. Diluted nEVs suspensions (1/200 in water) were illuminated by laser light and their movement under Brownian motion was captured for 60 s using a digital camera. Three videos were then analyzed using the Nanosight NTA 3.2 Analytical Software to provide nanoparticle concentrations and size distribution profiles.

#### 4.5.3. Western Blot Analysis

The same amount of extract nEVs and cells proteins (20 µg) were separated in 10% SDS-polyacrylamide gel for electrophoresis according their molecular weight. The proteins separated in gel were transferred onto PVDF membranes using a Trans-Blot Turbo System (Bio-Rad, Hercules, CA, USA). Nonspecific binding sites in the membrane were blocked for 1 h in TBS (Tris-buffered saline) containing 5% non-fat dry milk and 0.1% Tween 20. The blot was incubated overnight at 4 °C with primary antibodies: TSG101 (1/2500) (MyBiosource, San Diego, CA, USA), GAPDH (1/5000) (Santa Cruz) and calnexin (1/500) (Santa Cruz). After three washing steps with TBS-Tween 0.1%, the membrane was incubated during 1 h with the corresponding secondary antibody HRP-conjugated anti rabbit (1/5000) (Cell Signaling Technology) and anti-mouse (1/1000) (Cell Signaling Technology). The membrane blots were revealed with ECL substrate (Bio-Rad Laboratories, Inc., Hercules, CA, USA) and bands were analyzed using luminescent imaging system FluorChem.

### 4.6. Determination of Cellular and nEVs Proteins

Cellular and nEVs concentrations of BDNF, PRGN, NSE, APP, MMP9, ANGPTL-4, LCN2, PTX2, S100B, RAGE, DJ-1 and alpha synuclein were determined by a Luminex assay from R&D Systems, Inc.. Aβ_1-40_, Aβ_1-42_, pTau T181 and total tau levels were measured also with luminex assay from EMD Millipore Corp., (Burlington, MA, USA) according to the manufacturer’s instructions. Fifty microliters of each diluted sample were added to pre-coated beads with antibodies of interest. After the addition of biotinylated detection antibodies and phycoerythrin (PE)-conjugated streptavidin, beads were read using the Luminex 100/200 and data were analyzed using Xponent 4.2 software (Massachusetts, MO, USA) Concentrations of all markers in/on EVs and cells were normalized with the total protein amount. Assay sensitivities (minimum detectable concentrations in pg/mL) for all markers are presented in Table S1.

### 4.7. CML Determination

To measure CML levels in nEVs and SK-N-SH cells, sandwich ELISA provided by Cusabio Biothech Co., Ltd. was used according to the manufacturer’s instructions. CML values in nEVs and cells were normalized with the total protein amount. Assay sensitivity (minimum detectable concentrations in pg/mL) was 15.6 pg/mL (Table S1).

### 4.8. Statistical Analysis

Data were expressed as mean ± S.E.M using the SPSS or GraphPad Prism program. A *p* value less than 0.05 was considered statistically significant. For the clinical study, statistical analyses were performed using one-way ANOVA analysis followed by LSD test to test differences between groups. Receiver-operating characteristic (ROC) curves and the area under the ROC curve (AUC) were used to determine the ability of MG and GO serum levels to differentiate between the diseased and control populations. For the data obtained by nEVs and cell analyses, one-way ANOVA analysis followed by Dunnett’s test was used for the comparison between control and treated conditions.

## 5. Conclusions

The present study demonstrated that the serum levels of MG and GO increase in MCI patients and can be used as a peripheral marker for the diagnosis of this early stage of AD. Thus, cognitive decline associated with AD might be linked to an increase in MG levels due to the impairment of the dicarbonyls detoxification or production.

At the neuronal level, MG and GO affect neurotrophic, neuroprotective factors (BDNF, PRGN, NSE, APP and MMP-9) and modulator of inflammatory factors (ANGPTL-4 and LCN2) levels in neurons and nEVs. Our data suggest that targeting MG and GO may be a promising therapeutic strategy to prevent AD. From a clinical point of view, the reduction of MG and GO accumulation due to hyperglycemic conditions or impaired glucose metabolism, and the enhancement of dicarbonyls scavenging system may provide new therapeutic target opportunities to reduce the pathophysiological modifications related to carbonyl stress in AD.

## Figures and Tables

**Table 1:** Characteristics of control subjects, MCI and AD patients.

Parameters	Controls Subjects (*n* = 15)	MCI Patients (*n* = 16)	AD Patients		
			ES (AD) (*n* = 19)	MS (AD) (*n* = 14)	LS (AD) (*n* = 16)
Sex (M/F)	3/12	4/12	3/16	2/12	5/11
Age (years)	69.4 ± 1.17	73.63 ± 1.24 *	79.68 ± 1.2 ***	79.73 ± 1.3 ***	80.13 ± 1.6 ***
MMSE scores (/30)	29.67 ± 0.15	27.19 ± 0.47 *	23.79 ± 0.8 ***	21.73 ± 1.16 ***	ND
MoCA scores (/30)	27.53 ± 0.51	22.94 ± 0.79 **	17.06 ± 0.9 ***	12.55 ± 1.6 ***	ND
Glucose (mmol/l)	4.64 ± 0.15	4.95 ± 0.17	4.85 ± 0.13	5.3 ± 0.28 *	4.45 ± 0.13

Values are mean ± standard error of the mean (S.E.M). Statistical analysis was performed using the one-way ANOVA followed by LSD test with * *p* < 0.05, ** *p* < 0.01, *** *p* < 0.001 versus controls subjects. Abbreviations: AD, Alzheimer’s disease; ES, Early stage of Alzheimer’s disease; MS, Moderate-stage of Alzheimer’s disease; LS, Late-stage of Alzheimer’s disease; MMSE, Mini-mental state examination; MoCA, Montreal cognitive assessment; ND, Not detected.

**Table 2:** Cutoff values to separate MCI patients to control subjects and early and all AD patients.

Groups	Cutt off (nM)	Sensitivity (%)	Specificity (%)
Methylglyoxal [Controls subjects vs MCI patients]	>463.2	87.5	93.33
Methylglyoxal [MCI patients vs ES AD patients]	<545.1	63.16	62.5
Methylglyoxal [MCI patients vs AD patients]	<545.1	63.27	62.5
Glyoxal [Controls subjects vs MCI patients]	>652.2	68.75	80
Glyoxal [MCI patients vs ES AD patients]	<588.6	68.42	81.25
Glyoxal [MCI patients vs AD patients]	<605	67.35	81.25

## Supplementary Materials

Supplementary materials can be found at https://www.mdpi.com/1422-0067/20/19/4906/s1.

## Abbreviations

AD	Alzheimer’s disease
Aβ	Amyloid beta peptide
AGEs	Advanced glycation end products
ALDH	Aldehyde dehydrogenase
ANGPTL-4	Angiopoietin Like 4
APP	Amyloid precursor protein
BDNF	Brain-Derived Neurotrophic Factor
CEL	*N*-(carboxyethyl)-*L*-lysine
CML	*N*-(1-carboxymethyl)-*L*-lysine
CSF	Cerebrospinal fluid
ES	Early stage of Alzheimer’s disease
EVs	Extracellular vesicles
GO	Glyoxal
GAPDH	Glyceraldehyde 3-phosphate dehydrogenase
GSH	Reduced glutathione
GLO	Glyoxalase
GOLD	Glyoxal-lysine dimer
HPLC	High-performance liquid chromatographic
Icv	Intracerebroventricular
LCN2	Lipocalin-2
LS	Late stage of Alzheimer’s disease
LSD	Least significant difference
2-MQ	2-methylquinoxaline
5-MQ	5-methylquinoxaline
MCI	Mild cognitive impairment
MMP-9	Matrix metallopeptidase 9
MG	Methylglyoxal
MMSE	Mini-Mental State Examination
MoCA	Montreal Cognitive Assessment
MOLD	Methylglyoxal-lysine dimer
MS	Moderate stage of Alzheimer’s disease
NFTs	Neurofibrillary tangles
NSE	Neuron specific enolase
nEVs	Neuronal derived-extracellular vesicles
O-PD	*O*-Phenylenediamine
PRGN	Progranulin
PTX2	Pentraxin 2
RAGE	Receptor for advanced glycation end products
ROC	Receiver-operating characteristic curves
SSAO	Semicarbazidesensitive amine oxidase
TEM	Transmission electron microscopy
TSG101	Tumor susceptibility gene 101
