# Supplementary material for: Methylglyoxal and Glyoxal as Potential Peripheral Markers for MCI Diagnosis and Their Effects on the Expression of Neurotrophic, Inflammatory and Neurodegenerative Factors in Neurons and in Neuronal Derived-Extracellular Vesicles
Source: Int J Mol Sci. 2019 Oct 3;20(19):4906. doi: 10.3390/ijms20194906 (PMC6801730; doi:10.3390/ijms20194906)
Supplement: Supplementary file 1 [file ijms-20-04906-s001.docx]

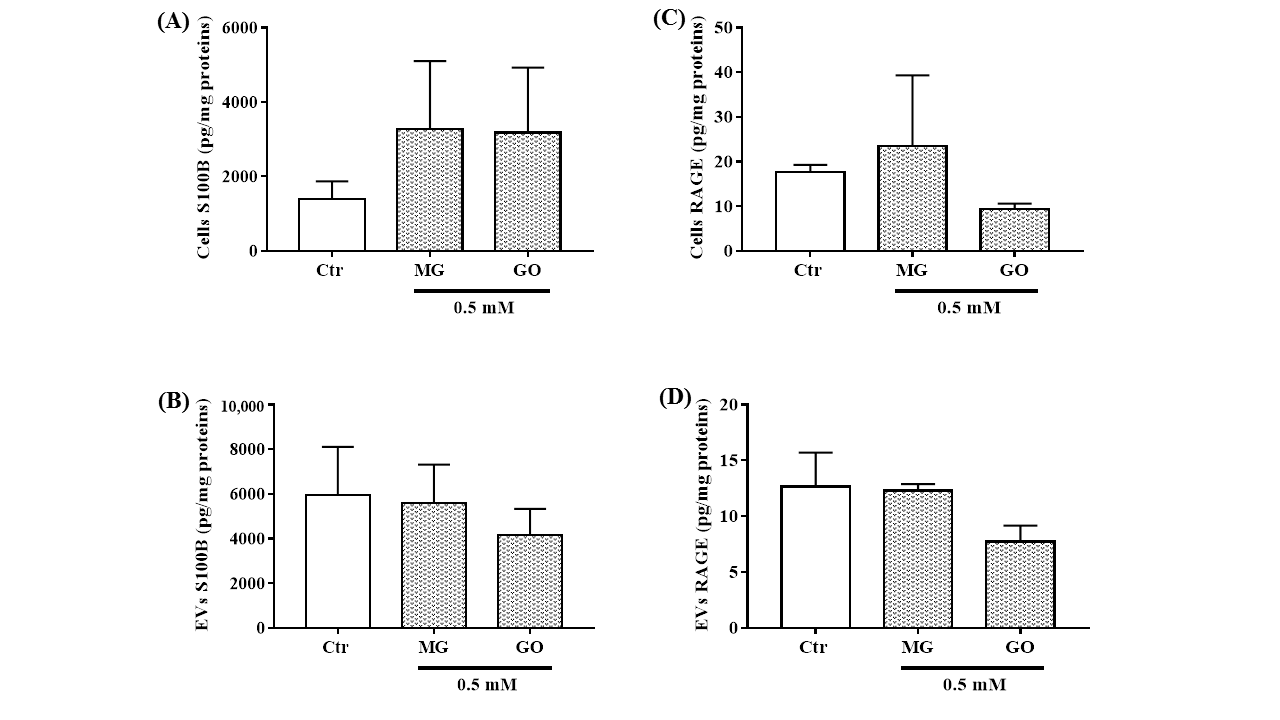


**Figure S1.** S100 B and RAGE levels in EVs and SK-N-SH controls cells and exposed to MG and GO. The level of each marker is normalized using total protein concentration. The graphed showed S 100B **(A and B)** and RAGE levels **(C and D)** in SK-N-SH cells and EVs. Values are mean ± S.E.M (from three separate experiments) and data groups were compared with one-way ANOVA followed by the Dunnett’s post hoc test.


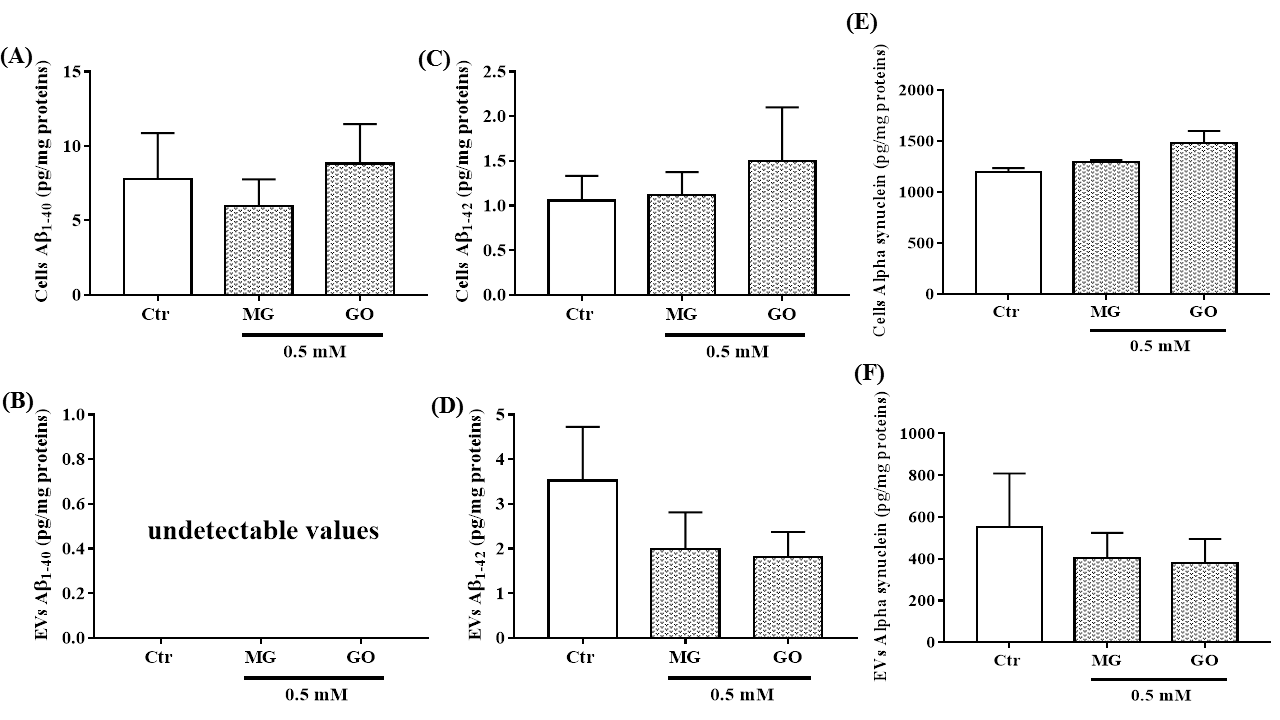


**Figure S2.** Aβ_1-40_, Aβ_1-42_ and alpha synuclein levels in EVs and SK-N-SH controls cells and exposed to MG and GO. The level of each marker is normalized using total protein concentration. The graphed showed Aβ_1-40_ **(A and B),** Aβ_1-42_ **(C and D)** and alpha synuclein levels **(E and F)** in SK-N-SH cells and EVs. Values are mean ± S.E.M (from three separate experiments) and data groups were compared with one-way ANOVA followed by the Dunnett’s post hoc test.


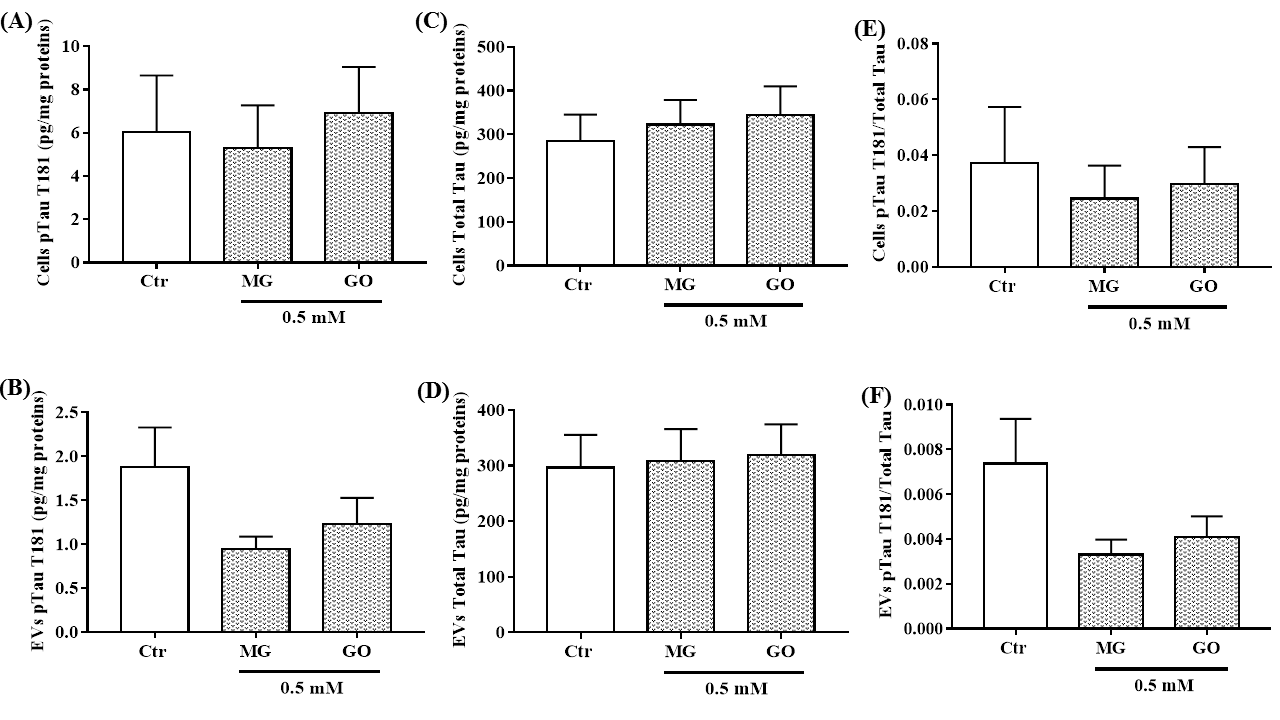


**Figure S3.** Phosphorylated tau T181 and total tau levels in EVs and SK-N-SH controls cells and exposed to MG and GO. The level of each marker is normalized using total protein concentration. The graphed showed ptau T181 **(A and B),** total tau levels **(C and D)** and ratio of ptau and total tau **(E and F)** in SK-N-SH cells and EVs. Values are mean ± S.E.M (from three separate experiments) and data groups were compared with one-way ANOVA followed by the Dunnett’s post hoc test.

**Table S1.** Assay sensitivities for all markers (minimum detectable concentrations) in pg/ml.

| **Markers** | **Assay sensitivity**  **(minimum detectable concentrations in pg/ml)** |
| --- | --- |
| **BDNF** | **0.32** |
| **PRGN** | **195** |
| **NSE** | **140** |
| **APP** | **349** |
| **MMP9** | **13.6** |
| **ANGPTL-4** | **86** |
| **LCN2** | **29.2** |
| **PTX2** | **1.66** |
| **S100B** | **4.34** |
| **RAGE** | **7.2** |
| **DJ-1** | **49.1** |
| **Alpha synuclein** | **5.16** |
| **Aβ_1-40_** | **6.2** |
| **Aβ_1-42_** | **1.3** |
| **pTau T181** | **0.7** |
| **Total tau** | **8.9** |
| **CML** | **15,6** |
